# Expression Profile Analysis Identifies a Novel Five-Gene Signature to Improve Prognosis Prediction of Glioblastoma

**DOI:** 10.3389/fgene.2019.00419

**Published:** 2019-05-03

**Authors:** Wen Yin, Guihua Tang, Quanwei Zhou, Yudong Cao, Haixia Li, Xianyong Fu, Zhaoping Wu, Xingjun Jiang

**Affiliations:** ^1^Department of Neurosurgery, Xiangya Hospital of Central South University, Changsha, China; ^2^Department of Clinical Laboratory, Hunan Provincial People’s Hospital (First Affiliated Hospital of Hunan Normal University), Changsha, China; ^3^Department of Operative Nursing, Xiangya Hospital of Central South University, Changsha, China

**Keywords:** glioblastoma, differentially expressed genes, gene signature, prognosis, TCGA, GEO

## Abstract

Glioblastoma multiforme (GBM) is the most aggressive primary central nervous system malignant tumor. The median survival of GBM patients is 12–15 months, and the 5 years survival rate is less than 5%. More novel molecular biomarkers are still urgently required to elucidate the mechanisms or improve the prognosis of GBM. This study aimed to explore novel biomarkers for GBM prognosis prediction. The gene expression profiles from The Cancer Genome Atlas (TCGA) and Gene Expression Omnibus (GEO) datasets of GBM were downloaded. A total of 2241 overlapping differentially expressed genes (DEGs) were identified from TCGA and GSE7696 datasets. By univariate COX regression survival analysis, 292 survival-related genes were found among these DEGs (*p* < 0.05). Functional enrichment analysis was performed based on these survival-related genes. A five-gene signature (PTPRN, RGS14, G6PC3, IGFBP2, and TIMP4) was further selected by multivariable Cox regression analysis and a prognostic model of this five-gene signature was constructed. Based on this risk score system, patients in the high-risk group had significantly poorer survival results than those in the low-risk group. Moreover, with the assistance of GEPIA http://gepia.cancer-pku.cn/, all five genes were found to be differentially expressed in GBM tissues compared with normal brain tissues. Furthermore, the co-expression network of the five genes was constructed based on weighted gene co-expression network analysis (WGCNA). Finally, this five-gene signature was further validated in other datasets. In conclusion, our study identified five novel biomarkers that have potential in the prognosis prediction of GBM.

## Introduction

Glioblastoma multiforme (GBM) is the most common and aggressive primary central nervous system malignant tumor with high morbidity and mortality. According to genomic abnormalities and gene expression, GBM can be divided into four molecular subtypes: classical, mesenchymal, neural, and proneural, which lay a foundation for understanding its inherent heterogeneity (Verhaak et al., 2010; Ma et al., 2018). In the United States, the incidence of GBM is 2.96 cases/100,000 population/year (Jhanwar-Uniyal et al., 2015). Although there are several treatment options, including surgery, radiotherapy and chemotherapy, the median survival of GBM patients remains 12–15 months, and the 5 years survival rate is less than 5% (Wen and Kesari, 2008; Ostrom et al., 2013).

With the development of next-generation sequencing technologies, many specific molecular signatures have been identified to better understand the molecular pathogenesis of GBM (Aldape et al., 2015). As a result, many potential diagnostic and prognostic biomarkers have been discovered that enable a more specific classification and a more precise outcome prediction of GBM. Some molecular markers including MGMT (O6-methylguanine DNA methyltransferase), IDH (isocitrate dehydrogenase), EGFR (epidermal growth factor receptor), and PTEN (phosphatase and tensin homolog) have been routinely tested in GBM patients clinically (van den Bent et al., 2017; Binabaj et al., 2018). More importantly, these molecular signatures have contributed to personalized therapeutic approaches and targeted anti-GBM therapies (Huang et al., 2017; Szopa et al., 2017). However, considering the poor prognosis of GBM, novel molecular biomarkers and new therapeutic strategies are still urgently required to elucidate the mechanisms of GBM or increase overall patient survival.

Previous studies have shown that gene expression profile analysis could detect gene signatures to predict the outcome for malignancy tumors (Luo et al., 2018; Mao et al., 2018; Zeng et al., 2018). Shergalis et al. (2018) discovered that 20 genes were overexpressed and correlated with poor survival outcomes in GBM patients by bioinformatics analysis using data from The Cancer Genome Atlas (TCGA) project. Bao et al. (2014) identified a nine-gene signature to predict the prognosis of glioma patients based on mRNA expression profiling from the Chinese Glioma Genome Atlas (CGGA) database. Therefore, it is necessary to understand the development and progression of GBM by identifying GBM-related genes and to investigate of their potential clinical roles and molecular mechanisms.

In this study, RNA-Seq data from TCGA and microarray data from the Gene Expression Omnibus (GEO) database of GBM were downloaded. Based on the overlapping differentially expressed genes (DEGs), the genes related to prognosis were screened. By using Cox regression, we developed a five-gene signature based risk score to demonstrate the association between gene expression and the prognosis of GBM. Moreover, we validated this signature in the GEO dataset and TCGA array dataset. These results might be able to provide new reference for the prognostic predication of GBM.

## Materials and Methods

### Data Source

The GBM RNA sequencing (RNA-seq) dataset and corresponding clinical follow-up information were downloaded from TCGA database (March, 2018). Subtype data of GBM were downloaded from UCSC Xena^1^. A total of 159 patients, including 154 samples of primary GBM patients and five samples of normal brain tissue were extracted for subsequent analysis.

Gene expression microarray data GSE7696 (Lambiv et al., 2011), including 71 samples of primary GBM patients and four samples of normal brain tissue, were downloaded from the National Center of Biotechnology Information (NCBI) Gene Expression Omnibus^2^. The dataset was based on the GPL570 platform of [HG-U133_Plus_2] Affymetrix Human Genome U133 Plus 2.0 Array (Affymetrix, Santa Clara, CA, United States).

### Differential Expression Analyses

Then, gene profiles were standard normalized within and among samples, respectively. Because the numerical distribution of RPKM (reads per kilo-base per million mapped reads) is too wide, the final expression level of a gene was defined as the log_2_(x + 1) of the raw expression level. Next, the DEGs between the tumor and normal samples were calculated by the limma package (Padj < 0.05 and | log_2_FC| > 1). The Venn diagram was produced by the VennDiagram R package (Chen and Boutros, 2011).

### Identification and Selection of Survival-Related Genes

Only the patients with detailed follow-up times were extracted for subsequent survival analyses. Univariate Cox regression survival analysis using the Survival package in R was performed to identify survival-related genes (Yang et al., 2016). Genes were selected with a *p*-value of less than 0.05.

### Go and KEGG Annotation of Survival-Related Genes

Gene Ontology (GO) enrichment and KEGG (Kyoto Encyclopedia of Genes and Genomes) analysis were performed on the survival-related genes (Ogata et al., 1999; Wanggou et al., 2016; Li et al., 2018). DAVID (The Database for Annotation, Visualization, and Integrated Discovery) (Dennis et al., 2003) software and the clusterProfiler package (Yu et al., 2012) in R were used to annotate and visualize GO terms and KEGG pathways.

### Gene Signature Identification and Risk Score System Establishment

Based on the top 100 survival-related genes in TCGA dataset, multivariable Cox proportional hazard regression analysis was performed to establish a risk score formula (O’Quigley and Moreau, 1986). As previously reported, a prognosis risk score formula could be constructed on the basis of a linear combination of the expression level (exp) multiplied by a regression coefficient (β) derived from the multivariate cox regression model.

$$\text{Risk Score}(RS)=\exp_{PTPRN}*\beta_{PTPRN}+\exp_{RGS14}*\beta_{RGS14}+\exp_{G6PC3}*\beta_{G6PC3}+\exp_{IGFBP2}*\beta_{IGFBP2}+\exp_{TIMP4}*\beta_{TIMP4}$$

Based on the formula, the risk score of each GBM patient was calculated, and then GBM patients were divided into high-risk score and low-risk score groups. The receiver operating characteristic (ROC) curve analysis was conducted using the R package “pROC.” After choosing an optimal cut-off point with the maximal sensitivity and specificity, the survival differences between the low-risk and high-risk groups were assessed by the Kaplan–Meier analysis with log-rank test. Similarly, to evaluate the predictive power of the five-gene signature in internal dataset, we assessed the gene signature within each subtype (classical, mesenchymal, neural, and proneural).

### Analysis in GEPIA and Exploring Co-expression by WGCNA

The expression levels of the five genes were acquired with the assistance of GEPIA^3^, which is a newly developed interactive web server for analyzing the RNA sequencing expression data of 23 types of cancers and normal samples from TCGA and the GTEx projects according to the standard processing pipeline (Tang et al., 2017).

To explore the regulatory network of the five genes, all the overlapped DEGs were analyzed by WGCNA (Ahn et al., 2016; Chen et al., 2018). Finally, the co-expression network of the five genes was constructed based on WGCNA and visualized by Cytoscape 3.6.1 (Shannon et al., 2003).

### Validation of the Five-Gene Prognostic Signature by the GEO Dataset and TCGA Microarray Dataset

Dataset GSE13041 from the GEO and TCGA microarray dataset were used to validate this five-gene prognostic signature (Lee et al., 2008). The GSE13041 dataset including 188 samples of GBM patients and the TCGA microarray dataset including 498 samples of GBM patients were both based on the Affymetrix Human Genome U133A Array platform (GPL97). The ROC curves and Kaplan–Meier analyses were used to validate the prognostic value of the five-gene for GBM patients.

## Results

### Differentially Expressed Genes (DEGs) in TCGA and GSE7696

**FIGURE 1:**
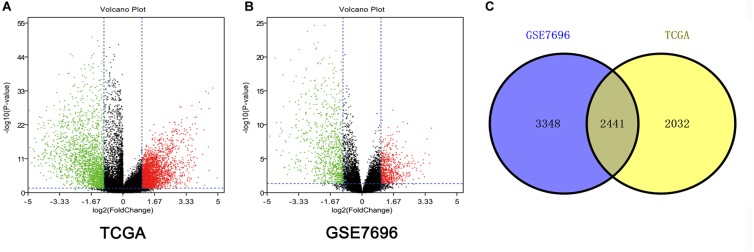
Identification of DEGs among TCGA and GEO datasets of GBM. **(A)** Volcano plots of DEGs in TCGA dataset. **(B)** Volcano plots of DEGs in GSE7696 dataset. **(C)** The Venn diagram of overlapping DEGs among TCGA and GSE7696 datasets.

Altogether, 4473 DEGs in TCGA dataset (Figure 1A) and 5789 DEGs in the GSE7696 dataset (Figure 1B) were screened by the limma package. The 2241 overlapping DEGs were screened for further analysis (Figure 1C).

### Survival-Related Genes in GBM

In TCGA dataset, every overlapped DEG was evaluated by univariate Cox regression survival analysis. Altogether, 292 significantly changed genes were considered -survival-related genes by the threshold of *p* < 0.05. The top 100 survival-related genes are shown in Supplementary Table 1.

### Go and KEGG Analysis of Survival-Related Genes

**FIGURE 2:**
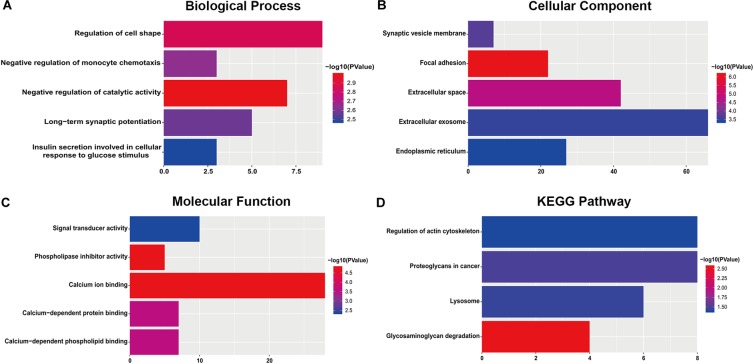
The most significantly enriched GO annotations and KEGG pathways of genes related to survival. The length of the bars represents the number of genes, and the color of the bars corresponds to the *p*-value according to legend. **(A)** Top 5 significantly enriched biological process. **(B)** Top 5 significantly enriched cellular component. **(C)** Top 5 significantly enriched molecular function. **(D)** Top 5 significantly enriched KEGG pathways.

For the “biological processes” (BP), negative regulation of catalytic activity, regulation of cell shape, negative regulation of monocyte chemotaxis, long-term synaptic potentiation and insulin secretion involved in cellular response to glucose stimulus were the commonly enriched categories (Figure 2A). For the “cellular component” (CC), the enriched categories were correlated with focal adhesion, extracellular space, synaptic vesicle membrane, extracellular exosome, and endoplasmic reticulum (Figure 2B). For the “molecular function” (MF), those genes mainly showed enrichment in calcium ion binding, phospholipase inhibitor activity, calcium-dependent protein binding, calcium-dependent phospholipid binding, and signal transducer activity (Figure 2C). KEGG pathway enrichment analysis suggested that glycosaminoglycan degradation was the most significant pathway. These genes also participated in following pathways: proteoglycans in cancer, lysosome, and regulation of the actin cytoskeleton (Figure 2D).

### Risk Score System Based on Five-Gene Signature

After multivariate Cox regression analysis was conducted for these 100 genes, five genes (PTPRN, RGS14, G6PC3, IGFBP2, and TIMP4) were selected as signature genes that can optimally predict the overall survival of patients with GBM (Table 1). To comprehensively investigate the association between these five genes and the prognosis of GBM, a five-gene survival risk score system was established based on their Cox coefficients.

$$\text{Risk Score}(RS)=0.50894*\exp_{PTPRN}+0.54671*\exp_{RGS14}+1.20753*\exp_{G6PC3}+0.25845*\exp_{IGFBP2}-0.20684*\exp_{TIMP4}$$

**Table 1:** Information about the five genes screened to build the risk score system.

Genes	Coefficient	HR	95% CI	*P*-value
PTPRN	0.50894	1.66353	1.4010–1.9753	6.35e-09
RGS14	0.54671	1.72757	1.2026–2.4816	0.00309
G6PC3	1.20753	3.34520	1.9960–5.6063	4.57e-06
IGFBP2	0.25845	1.29492	1.1096–1.5112	0.00104
TIMP4	-0.20684	0.81315	0.6951–0.9513	0.00976


**FIGURE 3:**
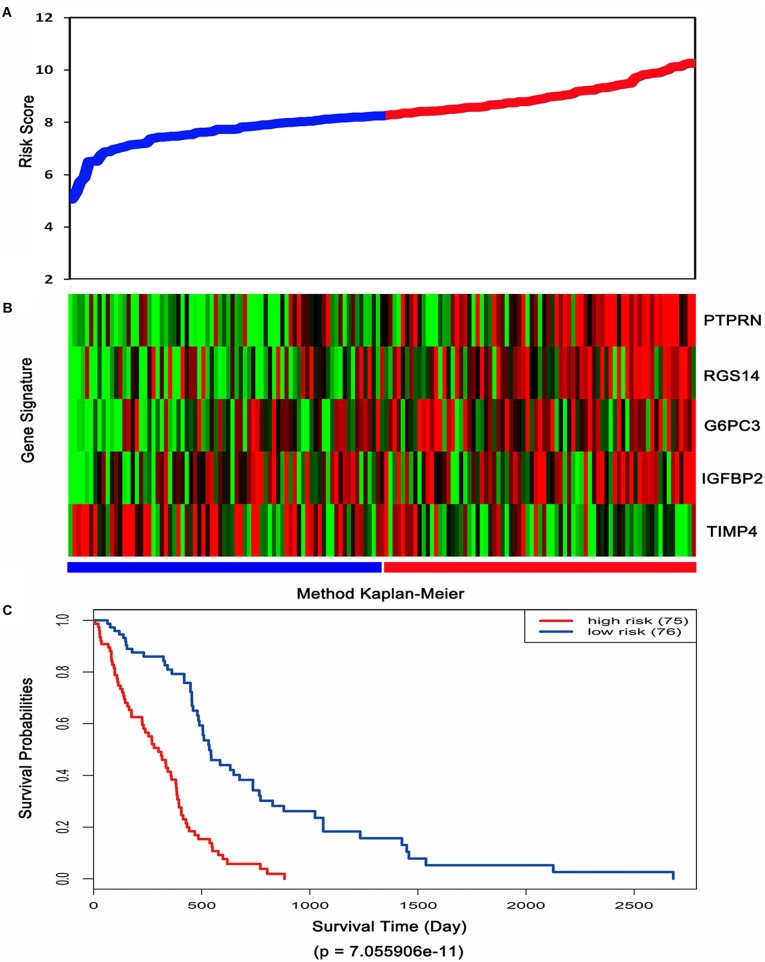
Risk score analysis, expression distribution and survival analysis of the five-gene signature in TCGA dataset. **(A)** The five-gene signature risk score distribution. **(B)** The heat-map of the five-gene expression profiles. Red indicates a higher expression and green indicates a lower expression. Blue bar: low-risk group. Red bar: high-risk group. **(C)** Kaplan–Meier analysis using the median risk score cut-off which divided patients into low-risk and high-risk groups.

Then, the risk score for each patient was calculated in TCGA dataset and ranked according to the risk scores. Thus, patients were divided into a high-risk group (*n* = 75) and a low-risk group (*n* = 76). The survival time of GBM patients was adversely associated with their risk scores (Figure 3A). A remarkably lower expression was noted for TIMP4 in the high-risk groups, while a higher expression was observed for the other genes in the high-risk groups (Figure 3B). The Kaplan–Meier analysis and log-rank test showed that patients in the low-risk group had a significantly positive overall survival time compared to the high-risk group (*p* = 7.055906e-11) (Figure 3C).

**FIGURE 4:**
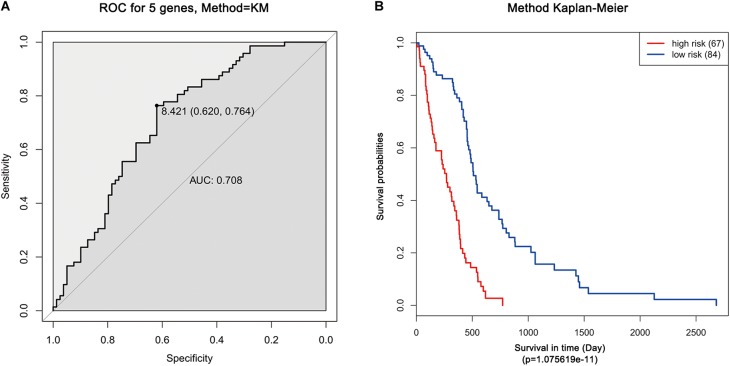
ROC and Kaplan–Meier analysis of the five-gene signature in TCGA dataset. **(A)** ROC analysis of the sensitivity and specificity of the survival time according to the five-gene signature based on risk score. **(B)** Kaplan–Meier analysis of the five-gene signature based risk score. Patients were divided into low-risk and high-risk groups based on the optimal cut-off point.

**FIGURE 5:**
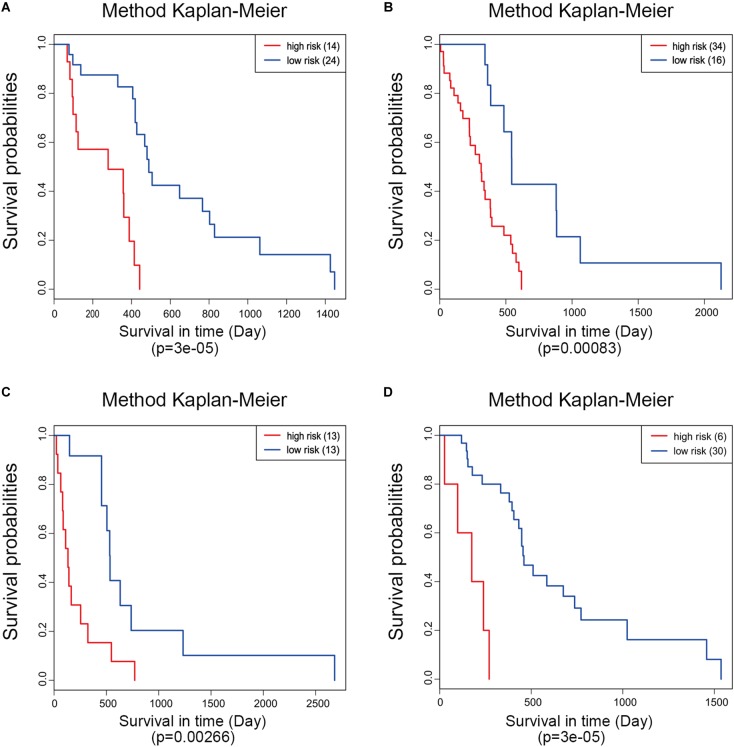
Kaplan–Meier analysis of the five-gene signature in different molecular subtypes of glioblastoma. Classical **(A)**, mesenchymal **(B)**, neural **(C)**, and proneural **(D)**.

Moreover, ROC analysis was performed for this risk score system. Figure 4A shows that the area under the ROC Curves (AUC) was 0.704. The optimal cutoff point was selected as 8.421. With this cutoff point, the patients were further divided into a high-risk group and a low-risk group. The Kaplan–Meier analysis and log-rank test further indicated a significant difference in overall survival between the two groups (*p* = 1.075619e-11) (Figure 4B). Similarly, with different cutoff points, the patients in each subtype were divided into a high-risk group and a low-risk group. The Kaplan–Meier analysis and log-rank test also indicated a significant difference between the two groups in each subtype (Figure 5A–D).

### Analysis in GEPIA and Exploring Co-expression by WGCNA

**FIGURE 6:**
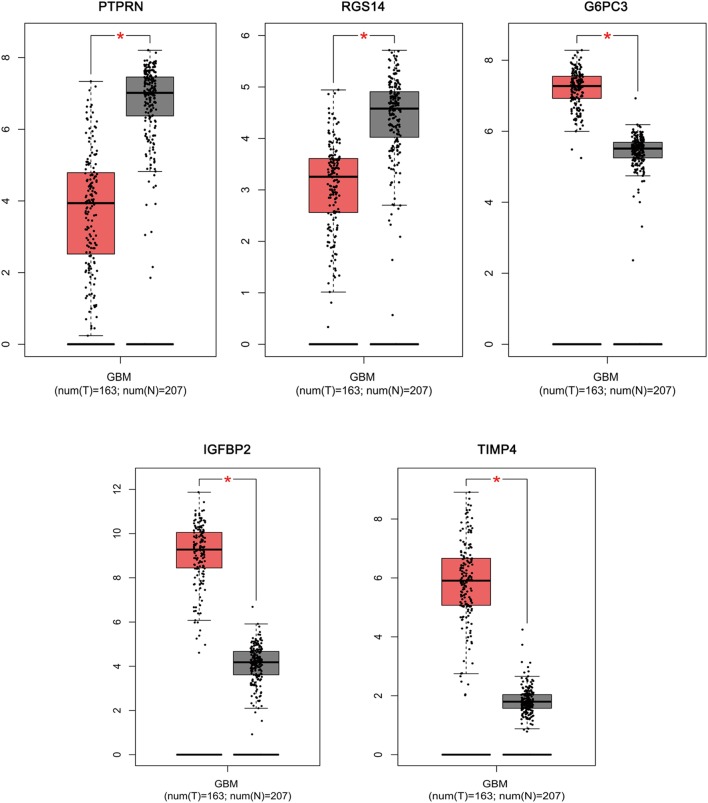
Comparisons of the expression of the five genes between GBM and non-GBM tissues in TCGA and GTEx based on GEPIA. The Y axis represents the log2 (TPM + 1) for gene expression. The gray bar indicates the non-GBM tissues, and the red bar shows the GBM tissues. These figures were derived from GEPIA. TPM: transcripts per kilobase million. ^∗^*p* < 0.05.

Based on the results derived from GEPIA, the expression of G6PC3, IGFBP2, and TIMP4 were significantly up-regulated in GBM, while the expression of PTPRN and RGS14 were significantly down-regulated (Figure 6). By using GEPIA, the selected five genes were verified as DEGs in GBM with amplified normal sample sizes.

**FIGURE 7:**
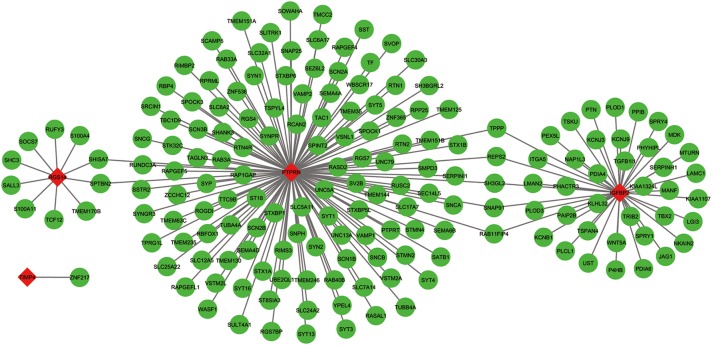
The co-expression network of the five-gene signature. Red diamonds showed the key genes and green nodes are genes which co-expressed with the key genes.

The co-expressed genes of the five genes were determined by WGCNA. Finally, 129 genes were discovered to be co-expressed with PTPRN, 41 genes were co-expressed with IGFBP2, 10 genes with RGS14 and 1 gene with TIMP4. However, no gene was co-expressed with G6PC3. The co-expression network of the four genes is visualized by WGCNA in Figure 7.

### Validation of the Five-Gene Prognostic Signature by GEO Dataset and TCGA Microarray Dataset

**FIGURE 8:**
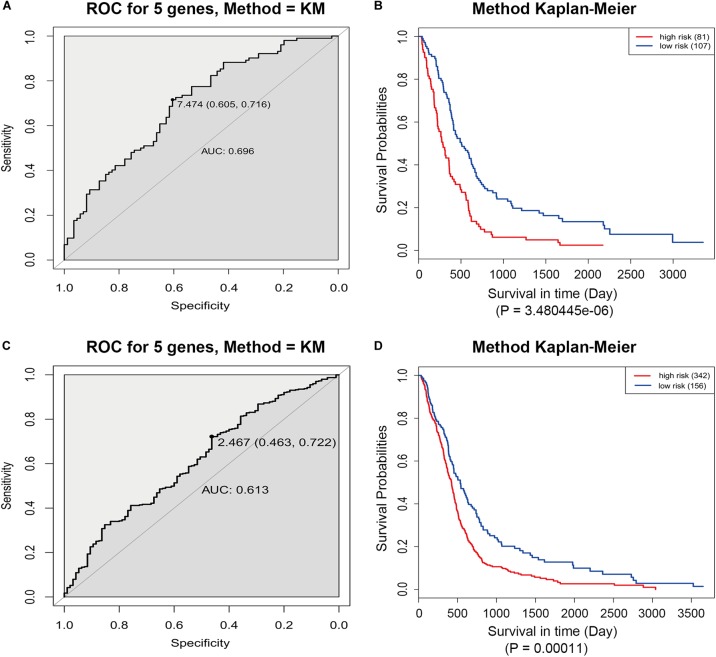
ROC and Kaplan–Meier analyses of the five-gene signature in validation datasets. **(A)** ROC analysis of the GSE13041 dataset. **(B)** Kaplan–Meier analysis of the GSE13041 dataset. **(C)** ROC analysis of the TCGA microarray dataset. **(D)** Kaplan–Meier analysis of the TCGA microarray dataset.

The GSE13041 dataset including 188 GBM patients and the TCGA microarray dataset including 498 GBM patients were used for the validation of the five-gene signature separately. Similarly, the risk score for each patient was calculated. ROC analyses were used to identify the optimal cutoff points (Figure 8A,C). Then, we divided the patients into a high-risk group and a low-risk group using the selected optimal cut-off points, respectively. The Kaplan–Meier analyses suggested a significantly prolonged survival time in the low-risk patients compared to that in the high-risk patients (*p* = 3.480445e-06 and *p* = 0.00011) (Figure 8B,D).

## Discussion

GBM is the most aggressive brain tumor associated with poor prognosis. By analyzing TCGA and GSE7696 datasets, we identified 2241 significantly overlapping DEGs. A total of 292 survival-related DEGs were selected from the overlapping DEGs. Functional analyses demonstrated that these genes are mainly associated with following pathways: glycosaminoglycan degradation, proteoglycans in cancer, lysosome, and regulation of the actin cytoskeleton. More importantly, based on multivariate Cox regression analysis of TCGA dataset, five genes which could predict overall survival were screen out, namely PTPRN, RGS14, G6PC3, IGFBP2, and TIMP4. According to their Cox coefficients derived from cox regression, a risk score system based on the five genes was established. Additionally, after identifying the optimal cut-off point by ROC analysis, patients were classified into high-risk and low-risk groups. This five-gene signature was further successfully validated as a prognostic marker in each subtype of GBM, another independent GEO dataset (GSE13041) and TCGA microarray dataset. Furthermore, differential expression analysis of the five genes in GEPIA validated that three genes (G6PC3, IGFBP2, and TIMP4) were significantly up-regulated and two genes (PTPRN and RGS14) were significantly down-regulated in GBM. Co-expression network analysis revealed the regulation network of the five genes. These results suggest that these genes may play an important role in the molecular pathogenesis, progression and prognosis of GBM.

Based on GO and KEGG enrichment analyses of the survival-related DEGs among different studies, “negative regulation of catalytic activity” was the most significant enrichment in BP. This indicated that inhibiting the catalytic activity of some genes may be critical for cancer progression. Coincidentally, Zhao et al. (2009) found that IDH1 mutation could inhibit IDH1 catalytic activity and contribute to the tumorigenesis of glioma. Other BPs such as regulation of cell shape and negative regulation of monocyte chemotaxis were also enriched. For the CC category, focal adhesion was the most significant enrichment which has been shown to be as a major determinant of cell migration and an essential process in tumor invasion (Garzon-Muvdi et al., 2012). The following three kinds of CCs, extracellular space, synaptic vesicle membrane and extracellular exosome, may also play important roles in tumor development and its micro-environmental manipulation (Wei et al., 2017). Regarding the MF category, calcium ion binding was the most affected MF. Ca^2+^-mediated cell connectivity and plasticity are unique features of the central nervous system, and the Ca^2+^/calmodulin-dependent process is able to regulate cell cycle progression and inhibit proliferation of malignant glioma (Cheng et al., 1995; Liu et al., 2011). For KEGG pathway enrichment analysis, glycosaminoglycan degradation was the most significant pathway. Extracellular proteoglycans play critical roles in driving oncogenic pathways in tumor cells and promoting critical tumor-microenvironment interactions (Wade et al., 2013). The other KEGG pathways, proteoglycans in cancer, lysosome, and regulation of actin cytoskeleton, were also closely related to oncogenesis (Liu et al., 2012; Terakawa et al., 2013; Wade et al., 2013).

The five-gene signature provides a wealth of potential biological and therapeutic information about GBM. PTPRN (protein tyrosine phosphatase, receptor type N), located on the long arm of human chromosome 2 (2q35) (Lan et al., 1996), is an integral transmembrane protein of dense core vesicles and plays an important role in the secretion of hormones and neurotransmitters (Xu et al., 2016). PTPRN has been confirmed to be negatively related to the survival of hepatocellular carcinoma patients and closely related to liver tumorigenesis (Zhangyuan et al., 2018). Moreover, the hypermethylation of PTPRN is also associated with shorter survival in ovarian cancer patients (Bauerschlag et al., 2011). A high expression of PTPRN in small cell lung cancer is associated with tumor growth and proliferation. Interestingly, Shergalis et al. also found that a high PTPRN expression is strongly associated with a poor prognosis in GBM patients, which was consistent with our finding (Shergalis et al., 2018). RGS14 is a member of the regulator of the G-protein signaling (RGS) protein family and is highly expressed in the caudate nucleus of the brain, spleen and thymus (Cho et al., 2005; Gerber et al., 2016). Previous study found that RGS14 is important for centrosome function, transcriptional regulation and stress-induced cellular responses (Cho et al., 2005). However, little work has been done to elucidate the role of RGS14 in cancer. Interestingly, PTPRN and RGS14 expressed at low levels in GBM tissue, but their increased expression was associated with poor prognosis. The reason may be that they have different functions in normal and tumor tissues. More work is needed elucidate their functions in GBM. G6PC3, namely, glucose-6–phosphatase isoform β, is a catalysis subunit of- G6PC (Gao et al., 2017). G6PC (glucose-6–phosphatase) is a key enzyme that regulates glucose homeostasis and glycogenolysis, which has been reported as a specific enzyme regulating proliferation and invasiveness in several tumors, such as liver, kidney and ovarian cancer (Gao et al., 2017). Furthermore, a previous study revealed that G6PC is a key enzyme regulating glioblastoma invasion (Abbadi et al., 2014). Our study demonstrated that G6PC3 was significantly up-regulated in GBM samples compared with normal brain tissue, and the high expression of G6PC3 was closely related to a poor prognosis in GBM patients. IGFBP2 (Insulin-like growth factor binding protein 2), an important member of the Insulin-like growth factor binding protein family, modulates cell growth, differentiation, migration, and invasion in neoplasms (Fukushima and Kataoka, 2007). IGFBP2 is involved in immunosuppressive activities and is a potential immunotherapeutic target for GBM (Cai et al., 2018). Our study confirmed that IGFBP2 was significantly up-regulated in GBM and predicted a worse outcome for patients, which was consistent with the previous study (Cai et al., 2018). TIMP4 is a member of tissue inhibitors of matrix metalloproteinases (TIMPs), which are involved in several processes of tumorigenesis including proliferation, migration, and invasion (Boufraqech et al., 2016). A high-expression of TIMP4 has been found in patients with breast, cervical, and prostate cancers, whereas a low expression has been observed in patients with pancreatic cancer (Boufraqech et al., 2016). Interestingly, our study found that TIMP4 was high-expressed in GBM patients, however, its high expression was associated with a good prognosis in patients with GBM. More work is also needed elucidate its functions in GBM. In summary, the five-gene signature not only is robust for predicting the overall survival for GBM, but also has promising practical value in the treatment of GBM.

There are some limitations in our work. First of all, there were only very limited normal samples included in our differential expression analyses, which might neglect some potential mRNAs. Moreover, the efficiency of the five-gene signature should be confirmed in more GBM patients. Furthermore, the molecular mechanisms how the five-gene signature affected the prognosis of GBM patients should be further elucidated by a series of experiments.

## Conclusion

In conclusion, our study identified five novel biomarkers that have potential for the prognosis prediction in GBM. Moreover, our findings provide new insights into the pathogenesis and prognosis of GBM.

## Author Contributions

WY and XJ conceived and designed the study. GT, QZ, YC, HL, XF, and ZW performed the analysis procedures. GT, WY, and XJ analyzed the results. WY and XJ wrote the manuscript. All authors contributed to the editing of the manuscript.

## Conflict of Interest Statement

The authors declare that the research was conducted in the absence of any commercial or financial relationships that could be construed as a potential conflict of interest.

## Footnotes

1 http://xena.ucsc.edu/

2 http://www.ncbi.nlm.nih.gov/geo/

3 http://gepia.cancer-pku.cn/
