# Supplementary material for: Expression Profile Analysis Identifies a Novel Five-Gene Signature to Improve Prognosis Prediction of Glioblastoma
Source: Front Genet. 2019 May 3;10:419. doi: 10.3389/fgene.2019.00419 (PMC6509566; doi:10.3389/fgene.2019.00419)
Supplement: Supplementary file 1 [file Table_1.DOCX]

**Supplementary Table 1 The top 100 survival related genes in TCGA dataset.**

| Gene names | HR | Cox p Value |
| --- | --- | --- |
| PTPRN | 1.432225383 | 1.11E-05 |
| PTPRN2 | 1.810672443 | 1.85E-05 |
| LOXL1 | 1.434322975 | 3.91E-05 |
| HPCAL1 | 2.012780478 | 9.35E-05 |
| AEBP1 | 1.301798599 | 1.39E-04 |
| HAS1 | 2.00373096 | 1.81E-04 |
| TSPAN4 | 2.173357244 | 2.45E-04 |
| STEAP2 | 1.662044978 | 3.42E-04 |
| EFEMP2 | 1.528239742 | 4.37E-04 |
| FAM174A | 2.83446654 | 4.51E-04 |
| ITPKA | 1.68443658 | 4.73E-04 |
| COL22A1 | 1.377770507 | 6.20E-04 |
| PDIA4 | 1.698236732 | 6.35E-04 |
| SEMA4F | 1.870137392 | 6.40E-04 |
| RGS14 | 1.626596981 | 7.11E-04 |
| FKBP9 | 1.46931593 | 7.88E-04 |
| G6PC3 | 2.24225549 | 8.23E-04 |
| STEAP3 | 1.337248928 | 8.34E-04 |
| TIMP1 | 1.267484864 | 8.62E-04 |
| SLC9A7 | 2.028235894 | 0.001149805 |
| LITAF | 1.917144672 | 0.001201448 |
| HSPB1 | 1.486757537 | 0.001210723 |
| RGS4 | 1.304154488 | 0.001228881 |
| PTX3 | 1.233818869 | 0.001243624 |
| SLC43A3 | 1.726747977 | 0.001323283 |
| MICAL2 | 1.660230305 | 0.001386801 |
| TCF12 | 0.650639883 | 0.001392794 |
| MTHFD2 | 0.651910193 | 0.001461997 |
| MDK | 1.396247459 | 0.001699311 |
| PXN | 1.946054489 | 0.001924793 |
| ZNF22 | 0.504995359 | 0.00192614 |
| MAP1LC3A | 1.49064783 | 0.00203353 |
| LYNX1 | 1.462699273 | 0.002090812 |
| FAM60A | 0.68564944 | 0.002618 |
| PGBD5 | 1.637377724 | 0.002749103 |
| KDELR2 | 1.765350889 | 0.003080082 |
| IKBIP | 1.806250386 | 0.003133437 |
| TMED9 | 1.950971776 | 0.004274829 |
| PLCH1 | 1.743234091 | 0.004320926 |
| CLEC4G | 2.557406513 | 0.004365966 |
| CCDC64 | 1.790737533 | 0.004461311 |
| NRXN3 | 1.779289138 | 0.004543309 |
| TOLLIP | 1.935273236 | 0.004700817 |
| ANKH | 1.623226687 | 0.004713222 |
| STX1A | 1.432804356 | 0.004715231 |
| TMEM60 | 1.817944253 | 0.004900008 |
| ANO4 | 1.651641605 | 0.004982029 |
| DMTN | 1.352673191 | 0.005058295 |
| SLC39A10 | 0.542671005 | 0.00508749 |
| ZDHHC12 | 1.579012637 | 0.005186195 |
| C10orf35 | 1.366543442 | 0.005205026 |
| PLOD3 | 1.499279174 | 0.005336972 |
| COMMD2 | 0.567821487 | 0.005356412 |
| SDR16C5 | 2.85480712 | 0.005568323 |
| GPC5 | 1.280062468 | 0.005606757 |
| CLEC4GP1 | 2.792488333 | 0.005637722 |
| ANXA2 | 1.296513496 | 0.00564432 |
| IGFBP2 | 1.203460637 | 0.005669315 |
| USP3 | 0.506459052 | 0.005692259 |
| PLK2 | 1.371106479 | 0.005808295 |
| SUSD5 | 0.763798329 | 0.006051615 |
| ITGA5 | 1.414678302 | 0.006201582 |
| MSN | 1.430746656 | 0.006236914 |
| KDELC2 | 1.471279495 | 0.006462554 |
| RCN1 | 1.643540001 | 0.00656614 |
| SYT5 | 1.365224064 | 0.006825105 |
| NELL1 | 1.409735228 | 0.006902359 |
| HS3ST2 | 1.303792632 | 0.006912369 |
| PAK1 | 1.632300691 | 0.00694761 |
| GREM2 | 2.097442612 | 0.00758702 |
| HSPA5 | 1.620443749 | 0.007630579 |
| YWHAG | 1.722803559 | 0.007651399 |
| ANXA2P2 | 1.324473289 | 0.007697084 |
| AACS | 2.652859664 | 0.007993005 |
| THEMIS | 5.090779692 | 0.008087295 |
| RAB11FIP5 | 1.691309137 | 0.008228004 |
| AGBL4 | 2.196296656 | 0.008322958 |
| SH2D5 | 1.642119975 | 0.008345571 |
| IQSEC2 | 1.554496247 | 0.008424985 |
| GRN | 1.563782286 | 0.008432805 |
| STAT4 | 2.348190212 | 0.008681639 |
| LRRC73 | 1.510530497 | 0.00870208 |
| KHDRBS2 | 0.547416776 | 0.008749506 |
| SLC2A10 | 1.382747489 | 0.008898634 |
| C1RL | 1.298106515 | 0.008938823 |
| TIMP4 | 0.826004845 | 0.008958264 |
| TMEM150C | 1.466644824 | 0.008976471 |
| GABRA4 | 2.172172878 | 0.00899425 |
| FAM50B | 1.364064187 | 0.009021034 |
| PLD3 | 1.673674838 | 0.009237604 |
| ANXA11 | 1.687442188 | 0.009404063 |
| C1orf226 | 0.716353138 | 0.00943649 |
| DNAJA4 | 1.567734215 | 0.009730931 |
| SCG5 | 1.354109371 | 0.009979432 |
| MYD88 | 1.657551148 | 0.010087037 |
| PCSK1 | 1.264919917 | 0.010177289 |
| EMP3 | 1.240306054 | 0.010291512 |
| PRKAR1B | 1.485096527 | 0.010300433 |
| PTPRU | 1.347247029 | 0.010432456 |
| PRNP | 1.629047401 | 0.010479803 |

HR, hazard ratio.
